# Supplementary material for: Yield and Rhizosphere Soil Environment of Greenhouse Zucchini in Response to Different Planting and Breeding Waste Composts
Source: Microorganisms. 2023 Apr 14;11(4):1026. doi: 10.3390/microorganisms11041026 (PMC10143665; doi:10.3390/microorganisms11041026)
Supplement: Supplementary file 1 [file microorganisms-11-01026-s001.zip › microorganisms-2317532-supplementary.docx]

**Table S1.** The basic physical and chemical properties of the test planting and breeding waste materials.

| **Raw materials** | **TN**  **(g·kg^−1^)** | **TP**  **(g·kg^−1^)** | **TK**  **(g·kg^−1^)** | **AN**  **(mg·kg^−1^)** | **AP**  **(mg·kg^−1^)** | **AK**  **(mg·kg^−1^)** | **SOM**  **(mg·kg^−1^)** |
| --- | --- | --- | --- | --- | --- | --- | --- |
| mushroom residue | 9.1 | 6.65 | 11.4 | 630.58 | 284.33 | 9253.33 | 470.31 |
| cow mature | 9.0 | 5.71 | 13.3 | 686.58 | 383.29 | 6786.67 | 510.76 |
| sheep mature | 8.99 | 6.61 | 11.8 | 658.58 | 304.87 | 9320.0 | 945.67 |
| corn straw | 10.04 | 7.79 | 15.09 | 546.58 | 385.61 | 8246.67 | 998.77 |
| tail vegetables | 11.09 | 5.41 | 139.6 | 971.25 | 391.25 | 13886.67 | 614.43 |

**Table S2.** The basic physical and chemical properties of different planting and breeding waste composts.

| **Treatments** | **TN**  **(g·kg-1)** | **TP**  **(g·kg-1)** | **TK**  **(g·kg-1)** | **AN**  **(mg·kg-1)** | **AP**  **(mg·kg-1)** | **AK**  **(mg·kg-1)** | **SOM**  **(g·kg-1)** | **pH** | **EC**  **(mS·cm-1)** |
| --- | --- | --- | --- | --- | --- | --- | --- | --- | --- |
| CK2 | 9.19 | 4.27 | 14.60 | 434.58 | 122.11 | 7596.67 | 227.57 | 8.14 | 4.81 |
| T1 | 8.09 | 3.40 | 15.35 | 429.92 | 143.29 | 8123.33 | 195.33 | 7.95 | 4.92 |
| T2 | 9.37 | 4.12 | 13.76 | 415.92 | 100.87 | 7013.33 | 132.75 | 8.07 | 5.33 |
| T3 | 9.19 | 4.38 | 11.68 | 476.58 | 93.33 | 6856.67 | 176.37 | 8.03 | 5.63 |
| T4 | 10.13 | 4.54 | 14.99 | 504.58 | 165.40 | 8860.00 | 394.45 | 8.25 | 6.35 |
| T5 | 10.17 | 4.27 | 10.11 | 411.25 | 96.54 | 7453.33 | 195.33 | 8.08 | 5.32 |
| T6 | 10.49 | 4.50 | 14.28 | 415.92 | 166.01 | 8240.00 | 244.64 | 8.13 | 5.91 |
| T7 | 11.34 | 4.86 | 13.77 | 555.92 | 161.93 | 9746.67 | 384.97 | 8.18 | 6.19 |
